# Supplementary material for: Development of a self-management intervention for stroke survivors with aphasia using co-production and behaviour change theory: An outline of methods and processes
Source: PLoS One. 2021 Nov 23;16(11):e0259103. doi: 10.1371/journal.pone.0259103 (PMC8610248; doi:10.1371/journal.pone.0259103)
Supplement: S1 File — (PDF) [file pone.0259103.s001.pdf]

S1 Supporting information. Example worksheet.

<u>**Who** might be **involved** in **supporting self-management?**</u>

**Speech** and language **therapists** 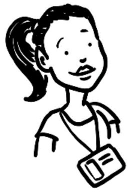	Pros	Cons
**Charity** support **workers** Stroke association	Pros	Cons
**Family** and **Friends** 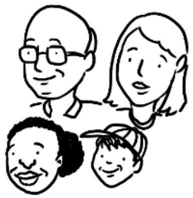	Pros	Cons
**Other:____________**	Pros	Cons

© University of Leeds and Bradford Teaching Hospitals NHS Foundation Trust

S1 Supporting information. Example worksheet.

When should **self-management** be **introduced?**

**Hospital** 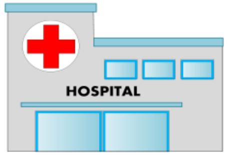	Pros	Cons
**Home** 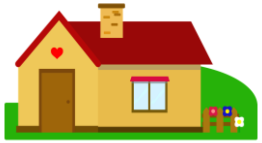	Pros	Cons
Review **appointments** 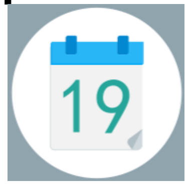	Pros	Cons
**Other:**_____________	Pros	Cons

Image credit: Images on this page reproduced with permission under licence by pixabay.com
