# Supplementary material for: Development of a self-management intervention for stroke survivors with aphasia using co-production and behaviour change theory: An outline of methods and processes
Source: PLoS One. 2021 Nov 23;16(11):e0259103. doi: 10.1371/journal.pone.0259103 (PMC8610248; doi:10.1371/journal.pone.0259103)
Supplement: S2 File — (PDF) [file pone.0259103.s002.pdf]

S2 Supporting information. Example infographic.

Self-management

**What** is **self-management**?	
**Living** with **stroke** and **aphasia** can be **challenging**	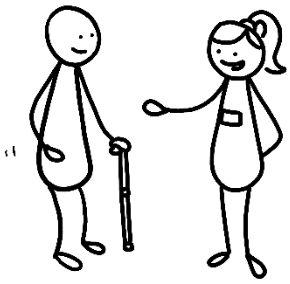
**Self-management** is **not** being **left** to do things on your **own**	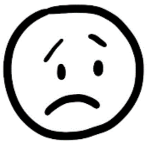
**But** finding ways **around** the **challenges** with the **support** of other **people**	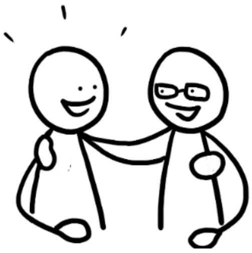
**So** that **stroke survivors** feel able to **cope**<br><br>and **do** the **things** they **enjoy**	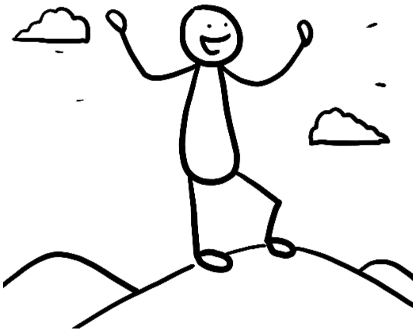
**Some** people say this is **finding** a **'new normal'**	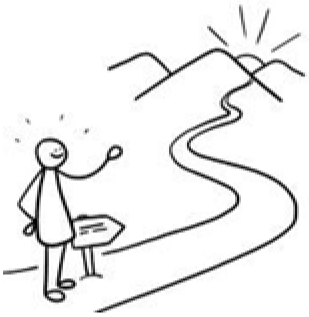

S2 Supporting information. Example infographic.

**Why** is **self-management important**?	
To increase **confidence**	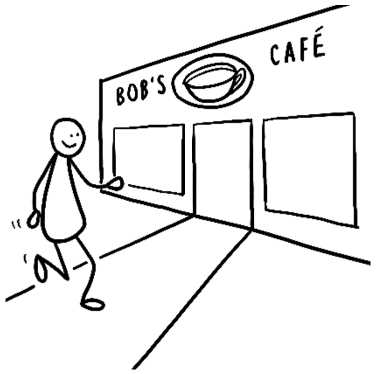
To help stroke survivors feel **in control** of their **lives**	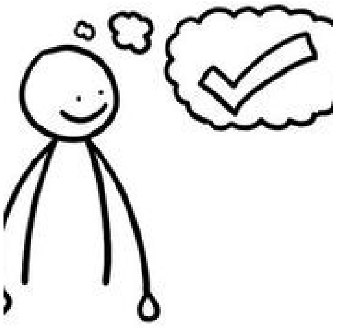
To **feel** a **sense** of **well-being**	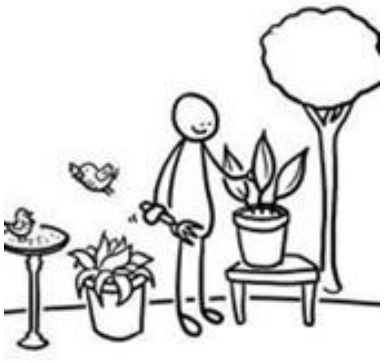

Sources: 1. https://twitter.com/clark_ev/status/826028658141888512?s=03

2. https://selfhelp4stroke.org/

Images: © University of Leeds and Bradford Teaching Hospitals NHS Foundation Trust
