# Supplementary figures and images for: Development of a self-management intervention for stroke survivors with aphasia using co-production and behaviour change theory: An outline of methods and processes
Source: PLoS One. 2021 Nov 23;16(11):e0259103. doi: 10.1371/journal.pone.0259103 (PMC8610248; doi:10.1371/journal.pone.0259103)

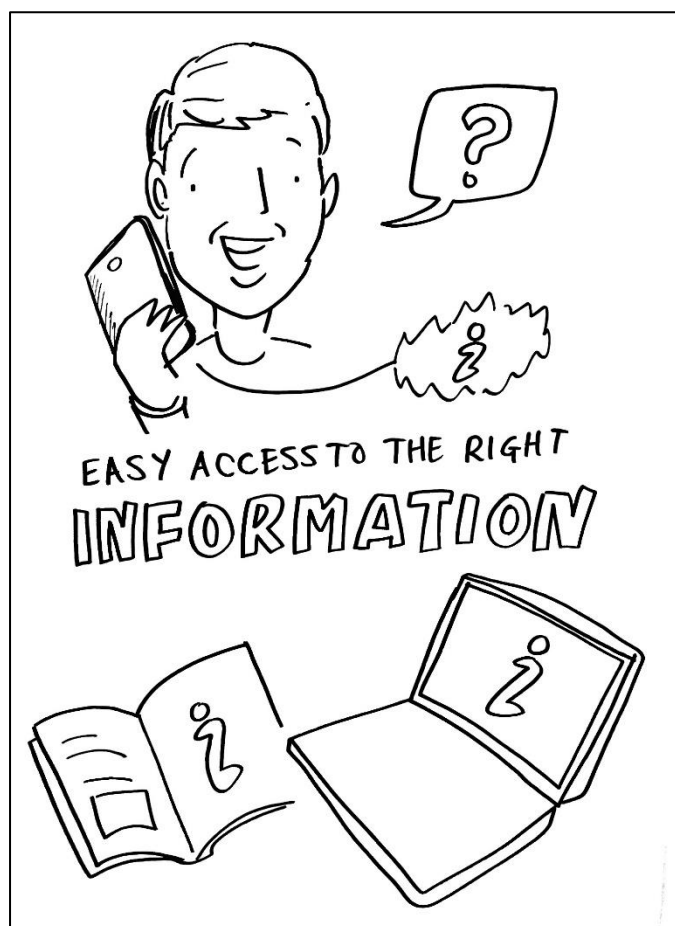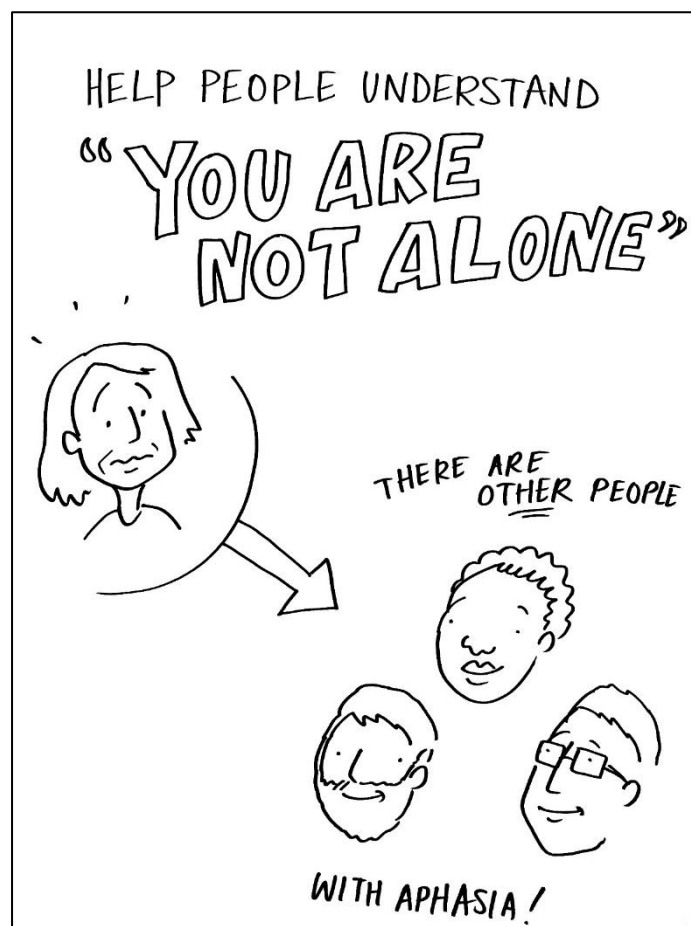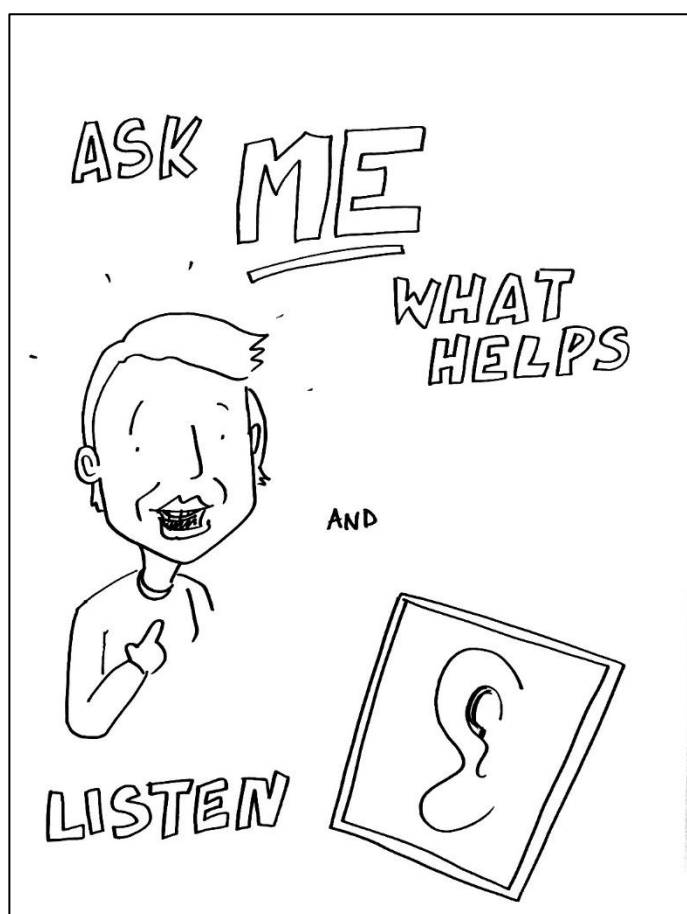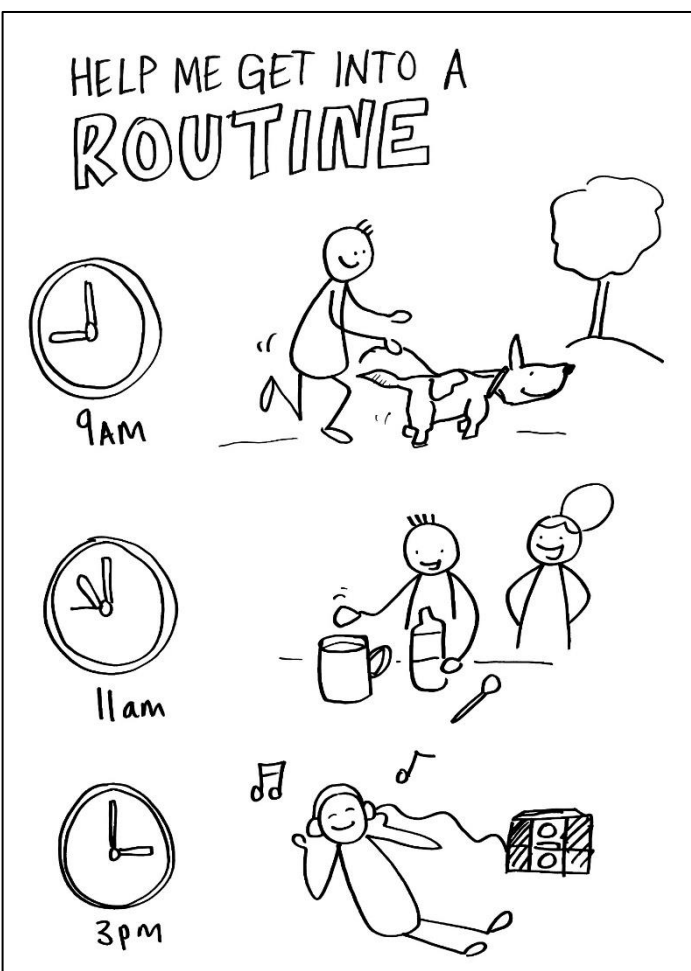

Supplement: S3 File — (PDF) [file pone.0259103.s003.pdf]

S4 Supporting information. Example of accessible summary.

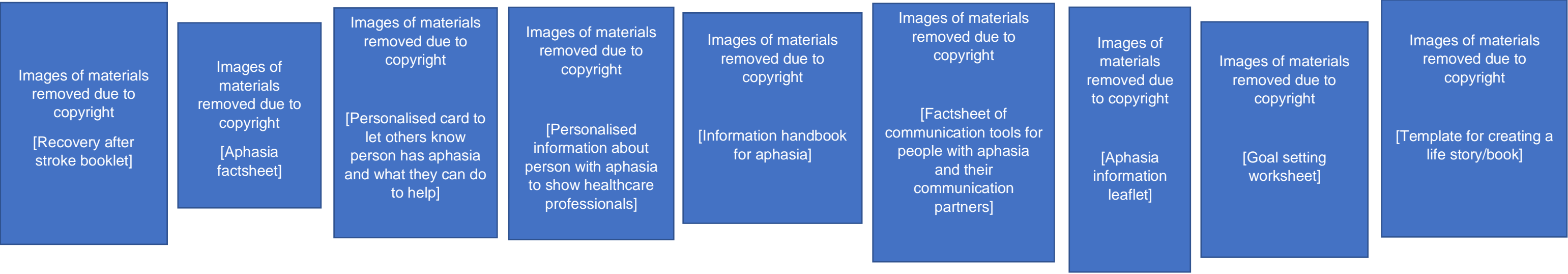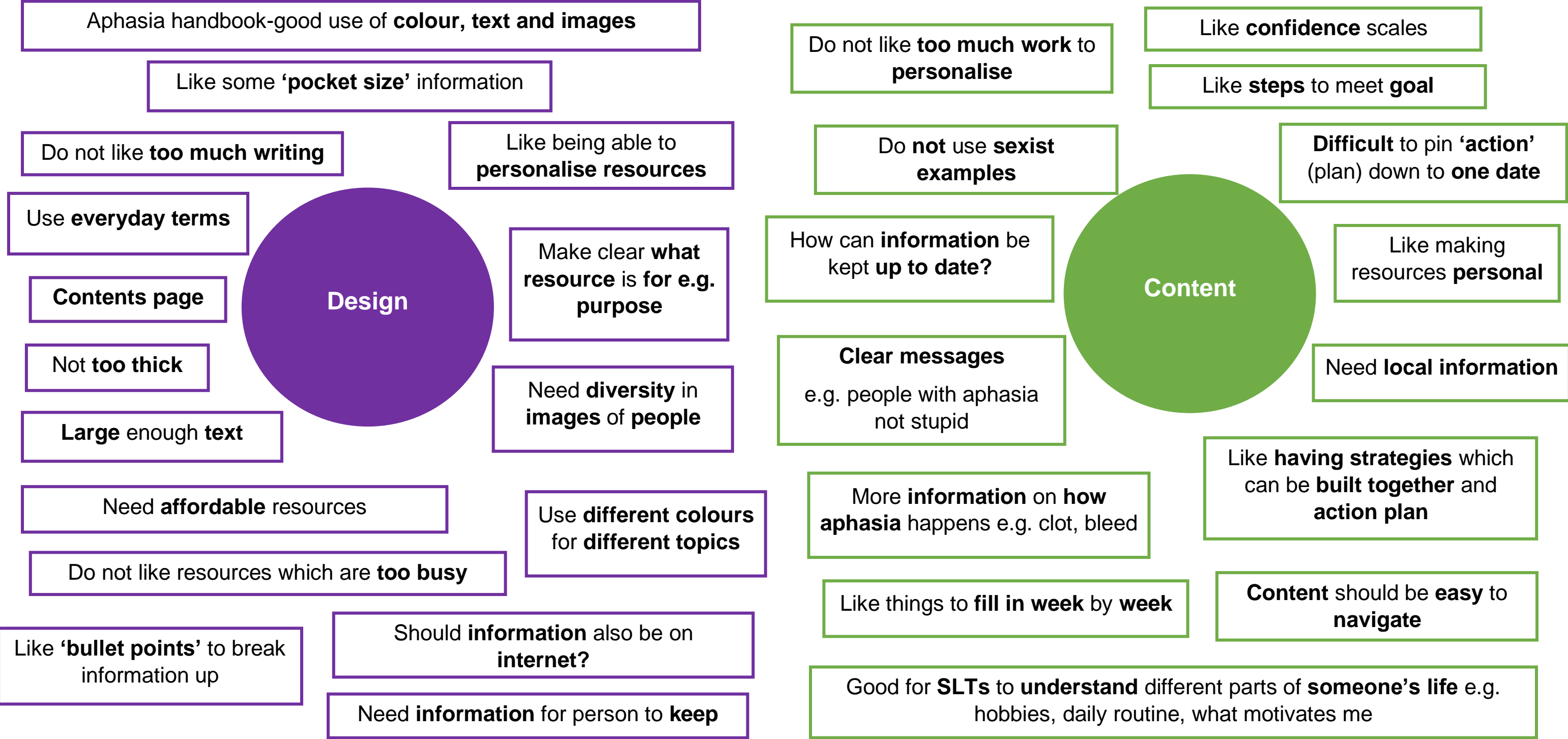

Supplement: S4 File — (PDF) [file pone.0259103.s004.pdf]
