# Supplementary material for: Development of a self-management intervention for stroke survivors with aphasia using co-production and behaviour change theory: An outline of methods and processes
Source: PLoS One. 2021 Nov 23;16(11):e0259103. doi: 10.1371/journal.pone.0259103 (PMC8610248; doi:10.1371/journal.pone.0259103)
Supplement: Attachment — Submitted filename: Response to reviewers.docx [file pone.0259103.s005.docx]

We wish to thank the reviewers for taking the time to read and comment on our manuscript and for their constructive feedback. Please see below the changes made to the manuscript in response to the comments provided.

| **Editorial** | |
| --- | --- |
| Comment | Changes made |
| 1. Please ensure that your manuscript meets PLOS ONE's style requirements, including those for file naming. The PLOS ONE style templates can be found at  <https://journals.plos.org/plosone/s/file?id=wjVg/PLOSOne_formatting_sample_main_body.pdf>  and  <https://journals.plos.org/plosone/s/file?id=ba62/PLOSOne_formatting_sample_title_authors_affiliations.pdf> | We have updated the manuscript to meet PLOS ONE’s style requirements including:   - Updating heading styles and size. - Double-spacing text. - Updating table labels. - Updating the reference style using the PLOS reference style in endnote. - Title page has been updated to include author contributions. - File names have been reviewed and revised. |
| 2. Thank you for stating the following in the Acknowledgments Section of your manuscript:  “The authors wish to thank The Stroke Association for providing the funding for this study.”  We note that you have provided funding information that is currently declared in your Funding Statement. However, funding information should not appear in the Acknowledgments section or other areas of your manuscript. We will only publish funding information present in the Funding Statement section of the online submission form.  Please remove any funding-related text from the manuscript and let us know how you would like to update your Funding Statement. Currently, your Funding Statement reads as follows:  “Funding for this study was provided by a postdoctoral fellowship awarded to FW by The Stroke Association (Ref: SA PDF 19\100011) (https://www.stroke.org.uk/research/support-programme-aphasia-life-after-stroke). Authors DC, MC and AF were co-applicants on this fellowship. The funder did not play a role in the design of the study, or in the collection and analysis of data, or in writing the manuscript, or in the decision to publish." Please include your amended statements within your cover letter; we will change the online submission form on your behalf. | Thank-you for pointing this out, we have removed reference to this in our acknowledgements. Please could the funding statement be updated as follows:  ‘Funding for this study was provided by a postdoctoral fellowship awarded to FW by The Stroke Association (Ref: SA PDF 19\100011) (<https://www.stroke.org.uk/research/support-programme-aphasia-life-after-stroke>). Authors DC, MC and AF were co-applicants on this fellowship. The authors wish to thank The Stroke Association for providing the funding for this study. The funder did not play a role in the design of the study, or in the collection and analysis of data, or in writing the manuscript, or in the decision to publish.’ |
| 3. Please include captions for your Supporting Information files at the end of your manuscript, and update any in-text citations to match accordingly. Please see our Supporting Information guidelines for more information: <http://journals.plos.org/plosone/s/supporting-information>. | Captions for supporting information are now provided at the end of the manuscript. In text citations updated to match accordingly. Supporting information file names have also been updated accordingly. |

| **Reviewer One** | |
| --- | --- |
| Comment | Authors response/changes made |
| 1. I would recommend to use the COREQ and SRQR (https://www.equator-network.org/reporting-guidelines) for reporting qualitative studies. Although there are items in these checklists, which are not relevant for this study, it can be used. Especially, the method section could be improved by providing more details on selection of the participants and data analyses. | We agree that some additional clarifications around the method would aid the reader in interpreting the results of the study. We also agree that not all points on the checklist are relevant as this paper reports a process for intervention development rather than a qualitative study (although we acknowledge there is some similarity in the methods used). We have detailed in the points you suggested where we have made changes and where we do not feel it is relevant to add any additional detail (and our reasoning for this). |
| 2. The discussion section could be improved by comparing the findings of current study to other research and theories. In addition, a broader implications of the research should be explained as well. | We have added a section in our discussion under the sub-heading ‘implications for self-management’. The section includes comparison of the findings of the current study with other stroke self-management interventions. The section also includes discussion of the theoretical underpinnings of previous self-management interventions and comparison with the theorical framework used in this study. We outline the broader implications of the research in:   - Highlighting the necessity of developing an aphasia specific self-management approach. - Developing an understanding of which components of self-management are effective and thus how such approaches should be integrated in the stroke pathway. |
| Minor and specific comments | |
| The title could be approved as follows: ‘’How to develop a complex self-care management intervention for aphasia for stroke survivors? A process description using co-production and behavior change theory’’. | Thank-you for this suggestion. We agree it would be useful for the reader to know from the title that this is a process paper. We have amended the title as follows: ‘Development of a self-management intervention for stroke survivors with aphasia using co-production and behaviour change theory: An outline of methods and processes.’ |
| Abstract  It is concise and clear, all relevant topics are addressed. | Thank-you. |
| Background   - Explain why partner-based and theory-based approach was chosen over other categories mentioned by O’Cathain et al. - The research question/aim should be stated at the end of the background section. - Discuss and explain: are there any comparable studies done using the same approach in different disease areas for the development of self-management interventions? If yes, what kind of development process did they follow? | - As outlined in the background section, there is no consensus about the best methods to develop a complex intervention and any of the approaches outlined by O’Cathain et al would be a valid approach to take. Our main rationale for using the approaches is the benefits we thought each approach brought (which are outlined on page 6). We have added additional clarification that the approaches are also advocated within established guidance for developing complex interventions produced by the MRC. - The aim is now stated in a separate section at the end of the background. - There is a very large literature on self-management in other long-term conditions. We felt it was beyond the scope of the introduction to describe this in detail and we were also mindful of keeping the introduction focused and succinct. We have signposted the reader to relevant development process papers in other disease areas on line 94 of the introduction (refs 22-24) and also signposted to a large literature review of self-management interventions in other conditions in the discussion (ref 62). |
| Methods   - Data availability; please explain and add information relevant to this topic. - Describe COAST and FAST tests in more details (lines 183-184) - Explain: ‘’NHS band 6 or above’’ (line 178) - For the following four topics, check reporting guidelines as well: -Regarding the topic ‘’Personal characteristics’’. Add section on this and describe all relevant characteristics of the authors or workshop leaders.   -Regarding the topic ‘’Participant selection’’: add more information related to this topic, e.g. -How were participants selected? -How many people refused to participate or dropped out? Reasons?  -Why did you not tried to include family members of patients with severe aphasia in the study as participation of this group of patients was too difficult?  -Regarding the topic ‘’Analyses of data’’ -How were the field notes analyzed? -How was coding done? By means of software or only manually?  Regarding ‘’data collection‘’ -Was data saturation a goal? - Recording? Face to face? Online workshop? | - We have reflected further on this point and carefully read the journal guidelines. Plos One requires the minimal data set to be available defined as ‘the data required to replicate all study findings reported in the article, as well as related metadata and methods’. We therefore believe we have included the relevant data required within the manuscript/supplementary files. - Further details have been added on the COAST and FAST tests including further details on items and scoring. - Further clarification of this term has been added. - Relevant personal characteristics of the workshop participants have been added on page 9. Further details of the facilitators experience of working with people with aphasia is described under the section ‘accessibility of workshops for stroke survivors with aphasia.’   -Further details on the eligibility criteria for the study have been added under the ‘participant’ section in the method. We have also clarified that SSWA/family members were a convenience sample and SLTs were purposively sampled to represent a mixture of acute/community services. We did not collect data on how many stakeholders were approached to participate and reasons for this have been added under this section (as recruitment occurred via gatekeepers). We have added additional details on participants who expressed an interest at the beginning of the results section and have clarified that no participants dropped out in the results section (sub-section attendance).    -We acknowledge that with hindsight we could have taken this approach. We have added this in the limitations section of the discussion section.  -We have clarified in the ‘data collection and analysis’ section of the methods that the evaluation of process is based on a summary of fieldnotes and that no formal analysis was undertaken.  - We do not think the concept of data saturation is applicable in this instance given that no formal qualitative data analysis was undertaken.  -We have clarified within the data analysis section that sessions were not audio recorded.  -We detail in the methods section under the sub-heading ‘intervention development groups’ that workshops 1-5 were held face-to-face and workshop 6 was held online due to COVID-19. |
| Results   - Were all family members relatives of the included patients? - Section intervention development (page 15 (row 341) to page 20 (row 503); although it contains a lot of relevant information; it is very long; try to give a brief summary of the different development stages and put the other details in an appendix. A figure containing a summary what was done in each step would be helpful for the reader. - A brief overview of the intended intervention should be presented. e.g. details on de modes of delivery and the description of different intervention components should be added. - Content table 3 is only explained very brief. E.g. indicate limited number of family members present in half of the workshops, give a reason for being absent. - Explain abbreviations in tables 2 and 4 - Explain briefly COM-B model using a footnote (page 18, line 426) | - Yes. We hope this point has been clarified by the additional information provided in the methods section re: our inclusion criteria for family members. - We have removed some examples from the text, however, feel that this level of detail is necessary for the process to be understood and/or replicated by other researchers. This level of detail was also positively noted by reviewer 2. Thank-you for the suggestion of adding a summary figure. We have added a summary of key outputs in relation to each intervention development stage outlined by the BCW in Fig 2. We hope this will aid the reader and signpost to sections within the results where they may wish to have further detail. - A brief description of the intervention is provided on pages 25 and 27 (track changes manuscript). On these pages we state the 4 main components of the intervention, intervention functions and mode of delivery.   As mentioned in the discussion, we have chosen not to fully describe the intervention to reduce the risk of contamination between intervention and control sites in a planned future cluster RCT. We are also mindful that the intervention may be refined after planned feasibility testing so do not wish to provide a definitive description at this point. However, we are open to reconsidering this and welcome the editors input on this point.   - Further detail has been added on the reasons for workshop absences. - Abbreviations explained in full have been added as footnotes to all tables. - The COM-B model is explained in the methods section under ‘overview of study design’. We hope the reader can refer back to this section and thus have decided not to duplicate this information in the results. |
| Discussion   - The sections discussing observations of current research could be grouped together in order to provide a clear overview of recommendations for future researchers which want to develop complex interventions in general and specific for aphasia (lines 558 to607, 623 to 630) - Discuss the impact of selection of a specific group of stakeholders for the generalizability of the intervention for all aphasia patients. - The analysis of the field notes was done primarily by one author; discuss this limitation in the discussion section. - Discuss whether the participation of not all stakeholders in every workshop may have influenced development process? | - Thank-you for this suggestion. We considered moving this section, however, felt it sat better as an overall reflection of the process taking in to consideration the benefits and challenges of using co-production and the BCW. We have added sub-headings in this section of the discussion to aid the reader. - A paragraph highlighting this issue and how we aimed to overcome this within our design has been added to the discussion under strengths and limitations. - This limitation has been recognised in a new paragraph (paragraph two) within the strengths and limitations section of the discussion. - As above, further discussion of this point has been added to paragraph two of the strengths and limitations section of the discussion. |

| **Reviewer Two** | |
| --- | --- |
| Comment | Authors response/changes made |
| The paper is well-written and deeply detailed. Readers, even from research different areas, are able to replicate the process used to co-produce the intervention. There are also a lot of examples useful to better understand the process. | Thank-you. |
| In the method section: add a diagram describing all the steps done (from the preparatory research to the development of the intervention) otherwise the reader get lost | Summary added in Fig 1. |
| In the method section: the paragraph "Integration of evidence from preparatory research" appear disconnected from the discourse, maybe it could be enclosed previously in the first method paragraph "Preparatory research" | Thank-you for this suggestion. We tried moving this section to the first paragraph, however, felt that it did not sit well here as it referred to the intervention development groups (which had not been explained in the methods at this point). We have moved this section to after the ‘content of workshops’ and hope you’ll agree it sits better here as it relates to content. |
| The self-management implications derived from the intervention development are not so clearly stated, maybe it would be better to stress this more in the discussion and conclusion sections. | A separate sub-section has been added to the discussion to highlight the implications for self-management interventions. An additional sentence has also been added to the conclusion to highlight the self-management implications of the study. |
